# A Trajectory-Based Method to Explore Reaction Mechanisms

**DOI:** 10.3390/molecules23123156

**Published:** 2018-11-30

**Authors:** Saulo A. Vázquez, Xose L. Otero, Emilio Martinez-Nunez

**Affiliations:** 1 Departamento de Química Física, Facultade de Química, Campus Vida, Universidade de Santiago de Compostela, 15782 Santiago de Compostela, Spain; saulo.vazquez@usc.es; 2 Unidade de Bioestadística, Facultade de Medicina, Universidade de Santiago de Compostela, 15782 Santiago de Compostela, Spain; xoseluis.otero@usc.es

**Keywords:** automated algorithm, molecular dynamics, graph theory, statistical rate theory, kinetics simulations

## Abstract

The tsscds method, recently developed in our group, discovers chemical reaction mechanisms with minimal human intervention. It employs accelerated molecular dynamics, spectral graph theory, statistical rate theory and stochastic simulations to uncover chemical reaction paths and to solve the kinetics at the experimental conditions. In the present review, its application to solve mechanistic/kinetics problems in different research areas will be presented. Examples will be given of reactions involved in photodissociation dynamics, mass spectrometry, combustion chemistry and organometallic catalysis. Some planned improvements will also be described.

## 1. Introduction

Theoretical studies of reaction mechanisms can greatly benefit nowadays by leveraging the surge of automated methods developed in the last few years [1,2,3,4,5,6,7,8,9,10,11,12,13,14,15,16,17,18,19,20,21,22,23,24,25,26,27,28,29,30,31,32,33,34,35,36,37,38,39,40,41,42,43,44,45,46,47,48,49,50,51,52,53,54,55,56,57,58]. The idea of these new computational protocols is to substitute human intervention by less error-prone and less tedious automated algorithms. The automated methodologies range from chemical heuristics to the use of artificial forces to boost chemical reactions.

Our group has contributed with the development of a method called tsscds [43,44,45,46,47], which is based on accelerated molecular dynamics (MD), as are some others [29,30]. In our trajectories, the bonds of the molecule(s) are broken/formed thanks to large amounts of energy placed in each normal mode/atom of the system [45]. The distinctive feature of tsscds compared to others is the primary target of the post-processing analysis: the search for transition states (TS) rather than minima. Additionally, having determined the TS of a given process, its rate can easily be determined using transition state theory (TST) [59,60,61,62]. Thus, finding the relevant TSs on a given potential energy surface (PES), as our method does, is a subject of fundamental importance in chemistry.

In tsscds, after completion of a trajectory, an algorithm named bond breaking/formation search (BBFS) [45] is employed to select good TS guess structures, which are then optimized using Eigenvector Following (EF) [63]. In particular, the adjacency matrix, which indicates whether pairs of atoms form a bond, is monitored along each trajectory to identify the atoms/bonds involved in all chemical reactions taking place. Then, for each of the selected candidates, a partial optimization is firstly carried out by freezing the atoms involved in the reaction. The partially-optimized structure is subsequently subjected to TS optimization using the EF algorithm. The resulting TSs are then connected with the minima using intrinsic reaction coordinate (IRC) calculations [64]. Finally, tsscds also features a Kinetic Monte Carlo [65] module that provides the desired kinetic information using the network of TSs and minima. The source code can be downloaded from: http://forge.cesga.es/wiki/g/tsscds/HomePage.

The method has been successfully employed to study reactions involved in combustion [66,67], photolysis [68,69,70], mass spectrometry [71] and organometallic catalysis [43]. The aim of this review is to go over several examples where tsscds is employed to either discover new mechanisms and/or to explain the experiments. For detailed comparisons among different methods for exploring reaction space, the reader is referred to two recent reviews [58,72]. Additionally, in the last section, some planned improvements to enhance the efficiency/efficacy or to expand the scope of tsscds will be described.

## 2. Method

The method tsscds has been recently put forward by one of the authors as an automated tool to discover reaction mechanisms [44,45]. The basic idea behind tsscds is to run accelerated MD simulations with the aim to break/form bonds within a few hundred femtoseconds. The simulations are called “accelerated” because the molecules experience breakage or formation of new bonds very rapidly thanks to large amounts of vibrational energy placed in each normal mode of the system. In particular, a range of vibrational energies of ~20–50 kcal/mol per normal mode is initially employed. However, this range is automatically adjusted to attain at least 60% reactive trajectories in the MD simulations. Although the default option is to excite all vibrational modes of the system (using microcanonical normal mode sampling [73]), the user can decide to heat only one part of the system selecting a few normal modes to be initially excited. The latter option can be particularly useful for large systems.

The trajectory results are then analyzed with a post-processing algorithm (named BBFS), which identifies geometries with partly formed/broken bonds. Those structures serve as TS candidates in subsequent transition state optimizations. As detailed below, BBFS is based on the adjacency matrix, a Graph Theory object that has been employed in other successful automated methods like the one developed by Zimmerman [16]. Similar ideas have also been recently employed to analyze changes in conformations occurring in MD simulations [74].

Once the TSs are optimized, a reaction network is constructed by computing the intrinsic reaction coordinates (IRCs) [64] connecting TSs with intermediates [64]. The method employs two levels of theory: semi-empirical and ab initio/DFT. The semi-empirical calculations are performed to run the MD simulations and to obtain approximate TSs structures, while a higher level of theory is used to re-optimize the TSs and run IRC calculations. Two different electronic structure programs are employed: MOPAC2016 [75] and Gaussian09 [76] for the semi-empirical and ab initio/DFT calculations, respectively.

Unlike other automated methods like GRRM [42], our methodology has been employed so far to study only the ground electronic state. This is in part due to the fact that, currently, the potential energy and gradients can only be calculated at the semiempirical level of theory. The following is a description of the graph-theoretic tools and kinetic models employed in our method.

### 2.1. Graph Theory

A number of graph theoretic tools are employed at various stages of the procedure to find transition states (TS), screen their structures and construct a reaction network. Specifically, the time dependence of the adjacency matrix A is employed to discriminate TS-like geometries along the trajectories. The elements of this matrix are defined as:

(1) $$a_{ij}=\{\begin{array}{c} 1\;\mathrm{if}\;r_{ij}<r_{ij}^{\mathrm{ref}}\\ 0\;\;\mathrm{otherwise} \end{array}$$

with $r_{ij}$ being the distance between atoms i and j, and $r_{ij}^{\mathrm{ref}}$ a reference value that sets the upper limit for the bond length between the pair; in practice $r_{ij}^{\mathrm{ref}}$ is taken 20% greater than the sum of the covalent radii of i and j [45]. Thus, for an N-atom system, A is a N×N symmetric matrix with zeros on its diagonal.

Additionally, a weighted adjacency matrix $A^{w}$ is also employed in tsscds, whose off-diagonal elements are defined as:

(2) $$a_{ij}^{w}=\frac{1-{\left(r_{ij}/r_{ij}^{\mathrm{ref}}\right)}^{n}}{1-{\left(r_{ij}/r_{ij}^{\mathrm{ref}}\right)}^{m}}$$

Values of 6 and 12 have been employed in previous work for n and m, respectively [44]. Matrix $A^{w}$ contains information on the 3D geometry of the molecule [77] and its eigenvalues and eigenvectors can be employed to construct the so-called SPRINT coordinates [77]. An important property of these coordinates is their invariance with respect to translation, rotation and permutation of atoms, which makes them good molecular descriptors in trajectory-based methods. SPRINT coordinates are employed in tsscds to remove redundant structures.

Another matrix employed to determine the number of fragments in the system is the Laplacian, which is defined as:

(3) $$L^{\left(w\right)}=D-A^{\left(w\right)}$$

where D is the so-called degree matrix [44], whose elements are defined as:

(4) $$d_{ij}=\{\begin{array}{c} \deg\left(v_{i}\right)\mathrm{if}\;i=j\\ 0\;\;\mathrm{otherwise} \end{array}\;\;$$

where the degree $\deg\left(v_{i}\right)$ of an atom counts the number of contacts. The superscript (w) on L and A indicates that the corresponding matrix can either be weighted or not. For a non-weighted graph, the lowest eigenvalue of the Laplacian $\lambda_{1}$ is always zero, and the total number of zero eigenvalues determines the number of fragments of the system. For a weighted graph, an upper threshold for $\lambda_{1}^{w}$ is employed to identify fragmented structures [44]. The smallest non-zero eigenvalue is called the spectral gap, which is a measure of the degree of fragmentation of the structure. Thus, a small value of the spectral gap is associated with structures presenting non-covalent bonds (like van der Waals complexes), which are usually of no interest in chemical dynamics and kinetics.

The invariance of the SPRINT coordinates upon atom permutation is very important for the analyses of trajectories, where scrambling of atoms is frequent, as stated above. However, since the identity of each atom is absent in the adjacency matrix, SPRINT coordinates are identical for two structures where two non-equivalent atoms swap positions. For that reason, another type of molecular descriptor, based on a modified (weighted or not) adjacency matrix, is employed in tsscds. This new matrix, denoted as $A_{Z}^{\left(w\right)}$, contains the atomic numbers $Z_{i}$ of the atoms on the diagonal:

(5) $$a_{Z,ij}^{\left(w\right)}=\{\begin{array}{c} a_{ij}^{\left(w\right)}\;\mathrm{if}\;i\neq j\\ 1+\frac{Z_{i}}{10}\;\;\mathrm{if}\;i=j \end{array}$$

The expression for the diagonal elements is chosen to provide values comparable to the off-diagonal ones. Most importantly, the eigenvalues of this new matrix are only invariant with respect to the permutation of like atoms, and it is widely employed in tsscds.

### 2.2. Kinetics Simulations

The kinetics module of tsscds calculates rate constants for all the elementary steps and solves the set of first-order differential equations that describe the time evolution of all species (usually known as chemical master equation).

The rate constants can either be obtained as a function of temperature or energy. In the former case, transition state theory is employed [59,60,61,62]:

(6) $$k\left(T\right)=\sigma \frac{k_{B}T}{h}{\left(\frac{RT}{p_{0}}\right)}^{\Delta n}e^{-\frac{\Delta G^{‡}}{RT}}$$

where σ is the reaction path degeneracy, T is the temperature, h is Planck’s constant, $\Delta G^{‡}$ is the free energy of activation, $p_{0}$ is 1 bar and Δn = 1 (0) for bimolecular (unimolecular) reactions. The reaction path degeneracy is calculated as $\sigma =\frac{m^{TS}}{m}$, where m and $m^{TS}$ are the number of optical isomers of the reactant and transition states, respectively [78].

By contrast, the microcanonical rate constants are computed according to RRKM theory [78]:

(7) $$k\left(E\right)=\sigma \frac{W^{TS}\left(E\right)}{h\rho \left(E\right)}$$

where $W^{TS}\left(E\right)$ is the sum of states at the TS, ρ(E) is the density of states at the reactant, and E is the excitation energy of the system. The sums and densities of states are evaluated by direct count of the harmonic vibrational states using the Beyer-Swinehart algorithm. Once all state-to-state rates are determined, the chemical master equation is solved using Kinetic Monte Carlo simulations [65].

## 3. Overview of the Applications of Tsscds

The tsscds methodology has been employed in our lab to elucidate reaction mechanisms involved in photodissociation dynamics, mass spectrometry, combustion and organometallic catalysis, and in this section, several examples of each type are reviewed.

### 3.1. Photodissociation Dynamics

The dissociation of molecules can be promoted by using a laser source, which is known as photodissociation. Although many photodissociations take place in excited states, important mechanisms may occur in the ground electronic state following internal conversion. One of the quantities of interest is the product yield, which is usually determined in the experiments. The understanding of the dissociation channels in organic compounds has greatly benefited from the interplay between photolysis experiments and computational studies [70,79,80,81,82,83,84,85,86,87,88,89,90,91,92].

In this section, we summarize the results obtained with our automated method for systems that have also been studied in photodissociation experiments, highlighting the most important conclusions. In particular, the dissociation channels of formaldehyde, formic acid, vinyl cyanide, acrolein, acryloyl chloride and methyl cyanoformate were studied with our tsscds methodology.

Formaldehyde was employed as a benchmark system to test tsscds. The system had been previously studied with other automated methods like the scaled hypersphere search [33] and the global reaction route mapping (GRRM) [35]. The results obtained with all algorithms are comparable, and the kinetically-relevant stationary points are found using any procedure.

The study of the dissociation channels of formic acid (CO_2_H_2_) with tsscds revealed the existence of a new TS for the water-gas shift reaction (WGSR: CO + H_2_O → CO_2_ + H_2_) [45]. By contrast, GRRM predicted three consecutive steps for the shortest path of the WGSR [35]. The discovery of the new TS is a consequence of the highly non-IRC [93] nature of the trajectories employed in tsscds [45]; in other words, IRC jumps are not uncommon events [94]. This exemplifies one of the advantages of using trajectory-based methods to discover new reactions: we are not restricted to unimolecular reactions and the only constrain to discover new processes is the molecular formula of the system. Additionally, the large amounts of vibrational energy put in the normal modes enhances configurational space sampling in tsscds, which permits the exploration of all types of reactions.

**Figure 1:**
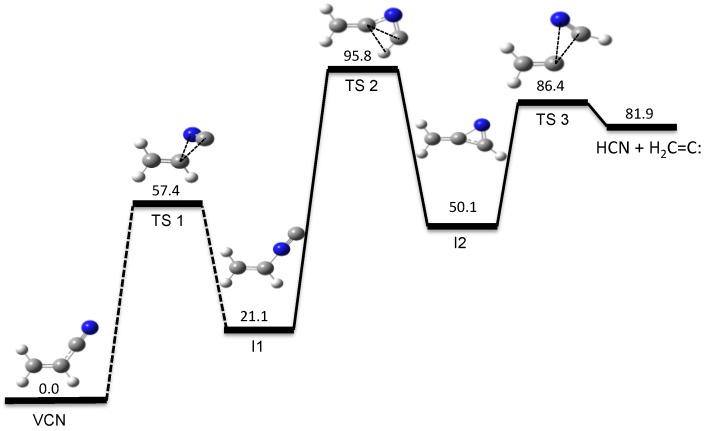
New HCN elimination mechanism from VCN obtained with tsscds. The numbers are relative energies (including the zero-point vibrational energy) with respect to VCN, calculated at the CCSD(T)/6-311++G(3df,3pd)//CCSD/6-311+G(2d,2p) level of theory with the vibrational frequencies obtained using CCSD/6-311+G(2d,2p) numerical Hessians.

Our automated computational study on the dissociation of vinyl cyanide (VCN) [70] provides a HCN/HNC branching ratio in nearly perfect agreement with experiments for an excitation energy of 148 kcal/mol [95]. Besides the traditional 3-center and 4-center elimination mechanisms found in many HX eliminations from CH_2_=CHX systems, a new HCN elimination pathway involving three TSs was discovered in the tsscds study. The new mechanism involves three TSs and two intermediates and is shown in Figure 1.

Although alternative routes for HX elimination were also found for other ethylene analogues, those pathways involved high-energy TSs and were not competitive with the conventional 3-center and 4-center channels. This was the first time a new HX elimination channel competes with the well-known 3-center and 4-center processes in the dissociation of CH_2_=CHX species.

**Figure 2:**
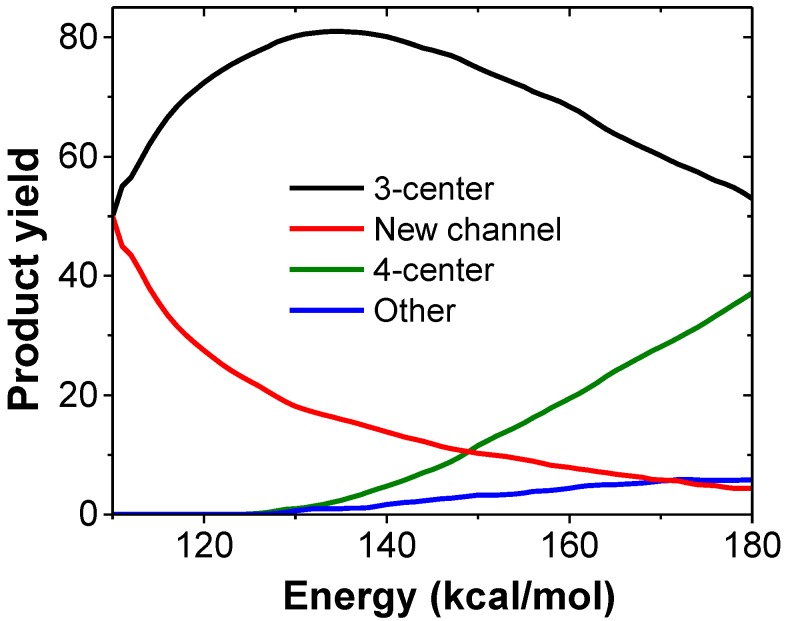
Kinetic simulation results of the different HCN elimination channels from VCN.

Figure 2 shows the product yields as a function of excitation energy obtained in our kinetic simulations from VCN. As seen in the figure, at low excitation energies (<150 kcal/mol) the new channel (red) is more important than the 4-center channel (green) and accounts for half of the HCN eliminations when the excitation energy is 110 kcal/mol.

The tsscds methodology was also employed to study the dissociation of acrolein (ACRL, C_3_H_4_O), which comprises many different fragmentation channels involving more than 250 transition states and 66 minima [44]. This system was studied with an enhanced procedure (now fully integrated in the method) consisting in the initialization of the MD simulations from multiple minima. In this new procedure the method works in an iterative manner. In the first iteration all MD simulations start from a starting structure, but once some TSs and intermediates are located, subsequent iterations utilize not only the starting equilibrium structure but also the newly generated intermediates to initialize the MD simulations. Compare to a single-minimum initialization, the use of multiple minima to start the dynamics ensures a better sampling of the PES of the system.

**Figure 3:**
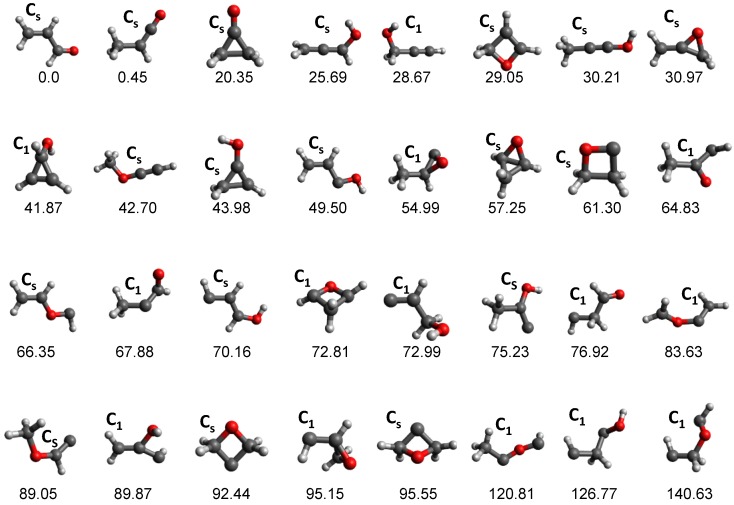
Minima obtained by tsscds for the C_3_H_4_O system. The structures are arranged in ascending order of their relative energies (shown at the bottom of each structure), which are obtained at the CCSD(T)/6-311+G(3df,2p)//B3LYP/6-311G(d,p) level of theory. Conformers are not included in the figure and only the lowest lying of each family is displayed.

The potential energy surface of the C_3_H_4_O system is very complex and the 32 equilibrium structures (not including conformers) shown in Figure 3 were found with tsscds, with ACRL being the global minimum. To exemplify the importance of automated reaction discovery methods, we compare our results with those obtained by Chin et al. [96], who manually located equilibrium structures and TSs. Using the same levels of theory as in our study, Chin et al. only found 6 of the 66 minima obtained with tsscds. Most importantly, the relative product abundances obtained with tsscds at 148 kcal/mol (the energy corresponding to the experimental wavelength of 193 nm) are much closer to the experimental results than the computational results of Chin et al., as seen in Table 1.

**Figure 4:**
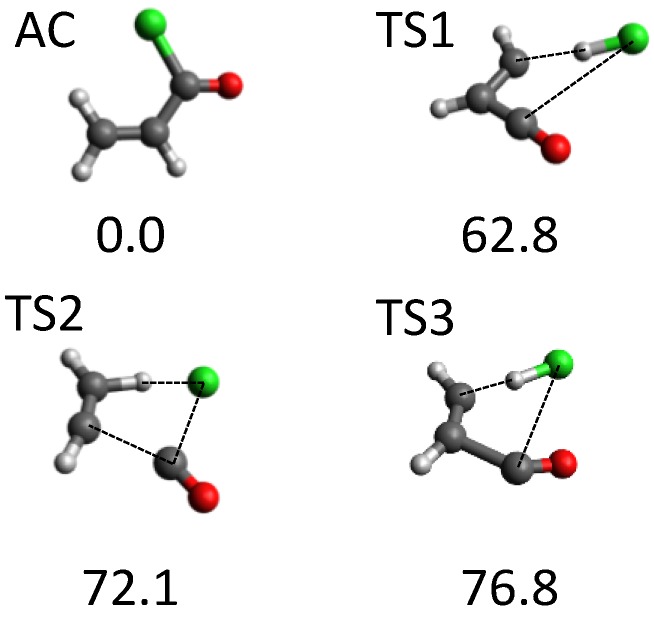
Structure of AC minimum and the three new TSs found with tsscds for the HCl elimination from AC. Numbers are relative energies in kcal/mol (including the zero-point vibrational energy) with respect to AC, calculated at the CCSD(T)/6-311+G(3df,2p)//B3LYP/6-311+G(2d,2p) level of theory.

Another system that attracted our attention was acryloyl chloride (AC). Overall, around 700 stationary points were found using our tsscds strategy. Of all possible dissociation channels from AC, experiments focus on the HCl dissociations. The use of our automated procedure led to the discovery of the three new HCl dissociation TSs [69] displayed in Figure 4; the figure also shows the AC equilibrium structure. The highest-energy TSs (TS2 and TS3) correspond to three-body dissociations leading to acetylene, carbon monoxide and hydrogen chloride, and they only become important at high excitation energies. By contrast, HCl elimination over TS1 is predominant at the experimental conditions (148 kcal/mol) [98], showing again that tsscds is capable of finding competitive pathways.

Finally, with the aim of exploring possible sources of HCN and HNC in astrophysical environments, the dissociation channels of methyl cyanoformate (MCF) were probed with tsscds, excited state calculations and photolysis experiments [68]. In particular, time-resolved infrared spectroscopy measurements indicate that both HCN and HNC are formed after the 193-nm photolysis of MCF [68]. The excited state calculations suggest that most of the dissociations take place in the S_2_ excited state leading to CH_3_O + NCCO via a Norrish type I reaction, in agreement with experiment. However, our calculations are also consistent with cascading internal conversion from S_2_ to produce vibrationally excited ground state MCF.

**Figure 5:**
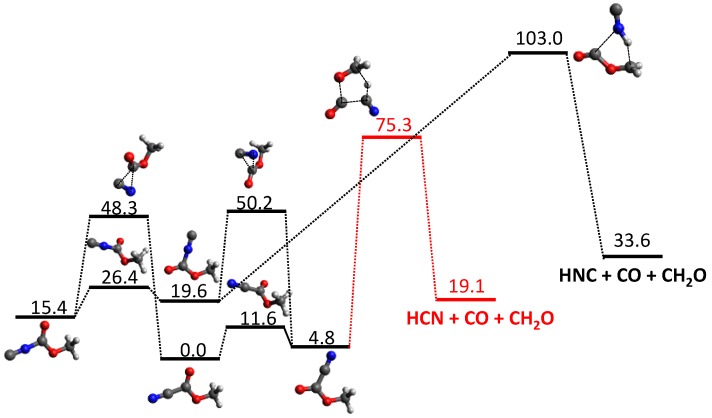
Relevant HCN and HNC pathways in the ground-state PES of methyl cyanoformate for an excitation energy of 148 kcal/mol. Relative energies (in kcal·mol^−1^) include ZPE contributions and were obtained by CCSD(T)/6-311++G(3df,3pd)//MP2/6-311+G(2d,2p) calculations.

To study the dissociation of vibrationally excited MCF molecules in the S_0_ electronic state, tsscds was employed. Our approach assumes that, after the internal conversion process, intramolecular vibrational redistribution is fast enough to ensure RRKM behavior. With the tsscds procedure several HNC and HCN mechanisms are found, and Figure 5 shows the kinetically-relevant ones at 148 kcal/mol. The kinetic simulations predict a HNC/HCN branching ratio of 0.01, which is in semiquantitative agreement with that determined in the experiments (≈0.07). The work provides further insights into the intriguing observation of overabundance of HNC in astrophysical environments.

### 3.2. Mass Spectrometry

The prediction of mass spectra remains much of a challenge for the community of computational chemists. The common computational approaches employed for such endeavor include statistical rate theory calculations, MD simulations and electronic structure calculations [99,100,101,102,103,104,105,106,107,108,109,110,111,112,113]. Our automated method is very useful in this regard and can easily be coupled with MD simulations of collisions to generate theoretically-based mass spectra as described below.

In particular, tsscds was employed to simulate mass spectrometry (MS) experiments of protonated uracil, [uracil]H^+^. Our computational results indicate that the decomposition of [uracil]H^+^ involves more than one thousand stationary points and 751 elementary reactions [71]. Branching ratios for the different fragmentation channels can be automatically obtained from tsscds. However, these fractions are a function of the ion’s internal energy and cannot be directly compared with MS experiments, where the collision energy in the center-of-mass framework ($E_{com}$) is employed instead. For that reason the tsscds results were combined with collisional dynamics simulations [71], which provide the fraction of $E_{com}$ transferred to the ion’s internal energy.

**Figure 6:**
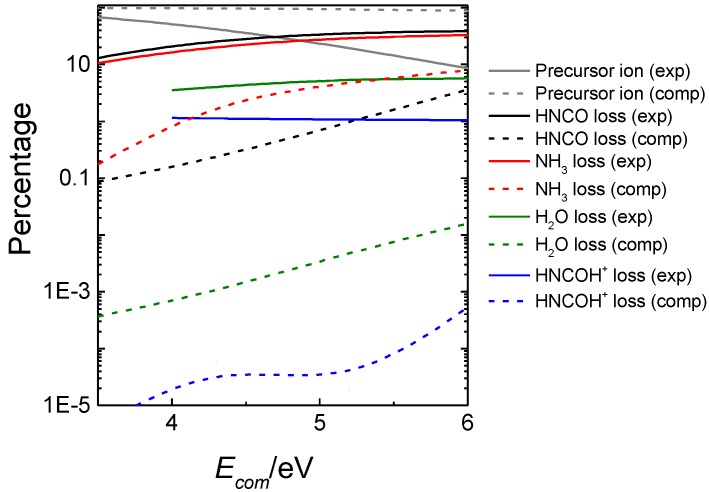
Experimental (exp) and calculated (comp) intensities of precursor and fragment ions produced in the fragmentation of protonated uracil.

The resulting computationally-predicted product abundances (dashed lines) are compared in Figure 6 with the experimental ones (solid lines). As seen in the figure, for the predominant dissociation channels, the computationally-predicted product abundances are in qualitative agreement with experiment, and formation of HNCO (black), NH_3_ (red), H_2_O (green) and HNCOH^+^ (blue) are the major channels. Discrepancies with experiment can be attributed to the possible existence of well-known non-statistical behavior in many collision-induced dissociations [100,114], which cannot be captured with our statistical model.

### 3.3. Combustion Chemistry

Modeling the combustion reactions of oxygenated fuels is of great interest due to their potential use as alternatives to conventional petroleum-based fuels. To investigate combustion mechanisms, it is important to use kinetic models and perform computer simulations as a complement to experimental determinations, due to the tremendous complexity of these chemical processes. In general, different approximations are employed in combustion simulations to handle the complicated mechanisms. One of these simplifications consist of considering only the lowest energy rotamers of the involved species, which can lead to large errors in the calculation of rate coefficients.

In a recent paper, our group analyzed the influence of multiple conformers and paths in the evaluation of rate constants and relative abundances of products formed in the thermal decomposition of 1-propanol radicals using different methodologies including tsscds [66]. Specifically, the most relevant pathways reported in the literature [115,116,117,118,119,120,121] are obtained with tsscds, except for the barrierless dissociation leading to propene + OH, since the present version of tsscds cannot handle this type of reactions. Of significance, an important number of reactant and TS conformers, not described in the previous studies, are obtained with tsscds.

**Figure 7:**
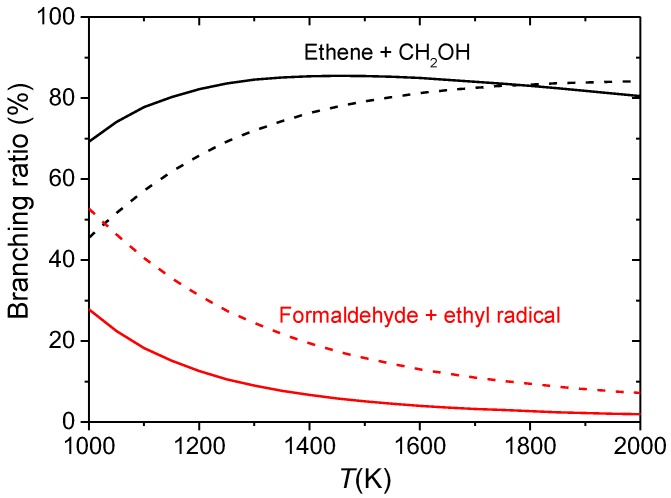
Branching ratios obtained in the kinetics simulations starting from one of the isomers of 1-propanol (only the two major mechanisms are shown). The solid and dashed lines correspond to the MP and 1W results, respectively.

A conformational reaction channel (CRC) was defined in our study [66] as the group of all the paths that connect the conformers of a given reactant with the corresponding TS conformers. The influence of these conformers on the rate constants and branchings ratios was investigated in detail [66]. To study such influence, the output of tsscds (families of CRCs) was fed into a computer program to treat torsional anharmonicity named Q2DTOR (also developed in our group) [122]. The results obtained with tsscds and Q2DTOR were finally employed to calculate variational transition state theory (VTST) [123,124,125] rate constants for all the CRCs. The multipath (MP) approach within VTST was employed [125,126,127,128,129], where the rate constant of a given CRC is calculated using contributions from all the conformers and paths. For comparison purposes the simplest one-well (1W) approach is also considered; in the 1W method only the most stable conformers of reactant and TS are considered. As seen in Figure 7, the product abundances obtained in the temperature range 1000–2000 K are greatly influenced by the selected approach (MP vs 1W), particularly for the major products: ethene + CH_2_OH and formaldehyde + ethyl radical [66]. Our results show the importance of using automated codes for discovering reaction mechanisms and sampling potential energy surfaces.

Very recently, Fenard et al. developed a detailed kinetic model of the low-temperature oxidation of tetrahydrofuran (THF) based on theoretically-calculated rate constants [67]. The reaction pathways involved in these processes were probed with our automated software tsscds [67] using CBS-QB3 as the choice for the high-level of electronic structure. The rate constants were determined using TST with a tunneling correction using an Eckart potential.

The predictions from the model developed by Fenard et al. are overall in good agreement with the different experimental measurements. Namely, it reproduces ignition delay times obtained in a rapid-compression machine and in a shock tube, as well as numerous product mole fractions measured in a jet-stirred reactor.

### 3.4. Organometallic Catalysis

Computational studies of organometallic catalysis are becoming increasingly more important because they can help elucidate reaction mechanisms, characterize catalytic intermediates, supplement experimental studies, and also because of their predictive power [124,130,131,132,133].

However, the traditional workflow of most computational studies consists of using chemical intuition in the design of reaction routes and construction of guess TS structures. In recent years the appearance of powerful automated computational methods to study homogenous catalysis [27,43,134,135,136] very much eased the tedious work of manual searches.

To exemplify the use of tsscds in organometallic catalysis, the cobalt-catalyzed hydroformylation of ethylene was chosen [43]. Very briefly, the first step in our computational study was to generate all combinations of the catalyst Co(CO)_3_ with any of the starting materials (CO, H_2_ and ethylene), which in this case amounts to eight. Each of these combinations has fewer atoms than the overall system and they were named sub-systems in our original paper [43]. Standard tsscds is then run in each sub-system to build the reaction networks. Finally, the full reaction network is obtained after merging the individual results for each sub-system.

**Figure 8:**
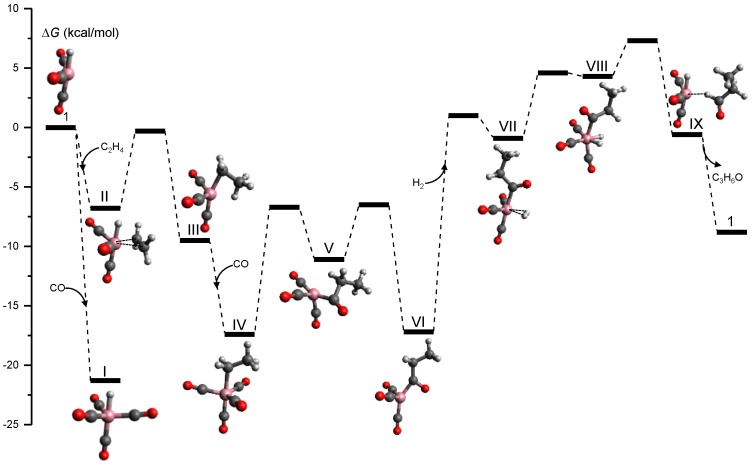
Free energy profile for the Co-catalyzed hydroformylation of ethylene obtained in our tsscds study using DFT calculations [133].

Figure 8 shows the tsscds-calculated free energy profile for the formation of propanal (C_3_H_6_O), which is the predominant channel; the level of theory employed was B3LYP/6-31G(d,p). As pointed out in the original paper, this is not the best electronic structure method for this system and it was only selected for comparison purposes. Additionally, we simulated the reactivity in the gas phase because, for this system, solvent effects are unimportant [43,133].

The mechanism shown in Figure 8 was obtained in an automated manner, and agrees with the one predict by Heck and Breslow in the 1960s [137] and with more recent mechanistic studies [133]. This is a very interesting result as we needed to make no assumptions in our automated calculations. Additionally, our method predicts that hydrogenation of ethylene is a side reaction that can be predominant under low CO partial pressures.

With the full reaction network constructed, the kinetics simulation module of tsscds can provide a rate law for the hydroformylation reaction when a range of different initial conditions for each species is employed. The kinetics calculations consist of transition state theory calculations [59,60,61,62] for the thermal rate constants at 423 K, and subsequent Monte Carlo simulations using different initial conditions of the reactants. Table 2 shows the orders of the catalyst and starting materials for the hydroformylation reaction obtained experimentally [138], with tsscds [43], using a kinetic model based on highly-accurate electronic structure calculations by Harvey and co-workers [133], and obtained from another automated method by Habershon [27].

As seen in Table 2, tsscds agrees rather well with experiment and with the results obtained by Harvey and co-workers [133]. Moreover, tsscds agrees much better with experiment than the other automated method does [27] (last column of Table 2), despite the fact that both employ the same alkene, initial conditions for the kinetics, and level of theory for the electronic structure calculations.

## 4. Improvements

In this section we describe some improvements we plan to implement in the near future. They include: the use of Spectral Graph Theory, implementation of knowledge-based methods, implementation of rare event acceleration MD simulations, interface with other electronic structure codes, reparametrization of semiempirical methods, and the study of condensed phase reactions.

### 4.1. Use of Spectral Graph Theory to Minimize the Number of Hessian Calculations

**Figure 9:**
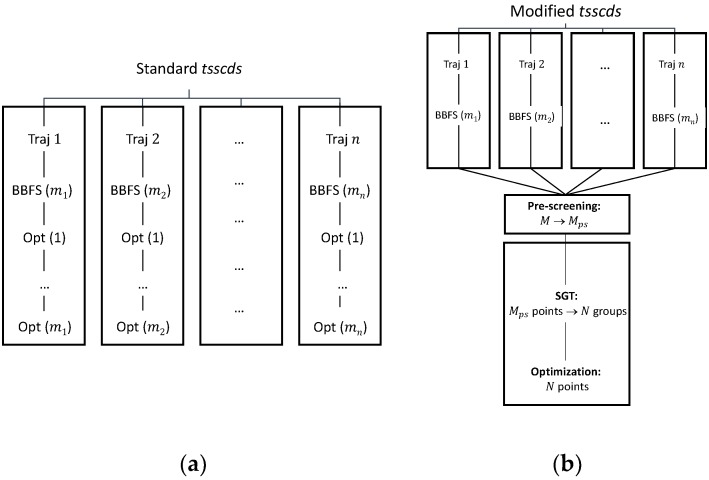
(**a**) Original tsscds showcasing an example with n different trajectories resulting in a total number of $M=\sum_{i=1}^{n}m_{i}$ optimizations. (**b**) Modified tsscds showcasing the same example as in panel (a) with n different trajectories resulting in a total number of N optimizations.

In standard tsscds, every single structure obtained after the BBFS analysis is subjected to TS optimization [45]. As seen in Figure 9a, for a trajectory i, BBFS selects $m_{i}$ TS candidates, which results in $M=\sum_{i=1}^{n}m_{i}$ optimizations, where n is the total number of trajectories. On the one hand, these M optimizations are the most CPU-time consuming step of the procedure as they involve Hessian calculations, while the integration of the trajectories only requires gradients. On the other hand, a number of those optimizations are repeated. This is so because trajectories visit more often those areas of the configurational space around the kinetically most relevant TSs, leading to multiple optimizations of those structures.

The workflow of the enhanced procedure is shown in Figure 9b. Briefly, instead of carrying out the optimizations for every single structure selected by the BBFS algorithm (as in the original implementation), the new procedure will run the MD simulations and store at once the M structures for the analysis of all trajectory data. This analysis will consist of a pre-screening, a Spectral Graph Theory (SGT) step, and the final optimization step.

Upon completion of the MD simulations, a pre-screening of the M structures will be performed based on the eigenvalues of the Laplacian matrix [44]. As pointed out above, the lowest eigenvalues of this matrix indicate the degree of fragmentation of the molecular system. We aim here to discard highly fragmented structures, i.e., TSs connecting van der Waals complexes, usually of negligible relevance in a kinetics study. In the SGT step the remaining points will be partitioned into N groups according to the eigenvalues of a TS adjacency matrix, calculated as the average of the reactant and product adjacency matrices. Finally, we will select the closest point (geometry) to the centroid of each cluster for optimization. With this new scheme the gain in efficiency can easily be quantified as the reduction in the number of optimizations from M to N.

### 4.2. Implementation of Knowledge-Based Mechanism Generators

A number of reaction discovery methods are based on the so-called chemical heuristics [23,48,49,50]. In these methods, molecules are typically represented as graphs, in pretty much the same way as in tsscds. Then, by applying transformations, based on encoded rules or principles inspired by organic chemistry, to the reactant molecule graph, reactions, products and intermediates can readily be obtained. Compared to MD-based methods, heuristic-based methods are less CPU-time demanding.

Our idea will be to combine a heuristic-based bias in the MD simulations alongside with our BBFS algorithm to obtain TSs. In particular, having defined a set of encoded rules based on chemical knowledge, every single MD simulation will suffer a different bias, aimed to trigger a particular reaction mechanism. In this way, the problem of multiple optimizations of a given TS mentioned above would be minimized, if not completely avoided. The bias (analytical) potentials will be added on top of the semiempirical potential to steer the dynamics towards a particular intermediate or product.

### 4.3. Implementation of Rare-Event Acceleration MD Methods

One of the shortcomings of tsscds is the fact that chemical reactions are triggered by using very high energies in the MD simulations. While this approach was successfully employed to tackle different problems, it is biased towards the entropically favored reaction pathways. To alleviate this drawback of the method we propose to replace the current MD strategy by the rare-event acceleration method named Boxed Molecular Dynamics (BXD) [139]. BXD has its roots in work done by one of us and D. Shalashilin more than a decade ago [140]. It introduces several reflective barriers in the phase space of a MD trajectory along a particular collective variable. Those boundaries are employed to push the dynamics along the collective variable into regions of phase space which would be rarely sampled in an unbiased trajectory. However, the use of BXD constrains in configuration space suffers from the same “entropic” bias mentioned above.

A generalization of BXD has been very recently put forward by Glowacki and co-workers [141]. They show that the BXD bias can also be introduced along the potential energy (E) of the system, which is referred to as BXDE. By scanning through potential energy “boxes”, the energetic “windows” at which different chemical reaction channels switch on or off can be identified. The software design of tsscds is highly modular, which means that interfacing it with BXDE only requires little effort, like the need of compatible input/output geometry formats in both codes and the use of extra keywords in tsscds.

### 4.4. Interface with Other Electronic Structure Codes

At present tsscds has been only interfaced with the MOPAC2016 [75] and the G09 [76] electronic structure packages. The MD simulation employs gradients calculated at the semiempirical level of theory, and the optimization step is carried out at both the semiempirical level with MOPAC2016 and using higher levels (ab initio/DFT) with G09. Although we plan to reparametrize a semiempirical Hamiltonian for use in organometallic catalysis (see below), we do not want to be limited to this low-level electronic structure calculations. Therefore, we will use the ASE package [142] to interface tsscds with other electronic structure codes like NWCHEM [143] or ORCA [144].

### 4.5. Reparametrization of Semiempirical Methods

The application of the tsscds method relies on the use of semiempirical Hamiltonians for exploring potential energy surfaces. For this reason, it is important that the semiempirical method provides a reasonably accurate representation of the system under investigation. Although significant improvements in these methods have been made over the last years [145], there are still known limitations, which claim for further developments and more accurate parametrizations. Two important limitations concern the non-covalent interactions for large systems and ligand dissociation energies for transition metal complexes. In both cases, the performance of the semiempirical methods is, in general, quite poor. Our goal is therefore to improve the description of both non-covalent interactions and transition metal complexes in PM7.

Regarding non-covalent interactions, we aim to develop an analytical correction for PM7. To this end, we will consider a set of small molecules, which are representative of the most important functional groups. All pairs of molecules will be considered to calculate interaction energies at three levels of theory: coupled-cluster (CC), DFT and PM7. For every pair, various orientations will be considered, each one emphasizing a different two-body interaction.

Then, sums of two-body Buckingham potentials (supplemented with damping functions for the dispersion) will be fit to the CC, DFT and PM7 interaction energies using our genetic algorithm program GAFit [146]. Finally, the resulting potentials $V_{\mathrm{fit},\mathrm{CC}}$, $V_{\mathrm{fit},\mathrm{DFT}}$ and $V_{\mathrm{fit},\mathrm{PM}7}$ will be employed to build corrections $V_{X}^{corr}$ to the PM7 interaction energies:

(8) $$V_{X}^{corr}=V_{\mathrm{fit},X}-V_{\mathrm{fit},\mathrm{PM}7}$$

where X is either CC or DFT. Whereas the $V_{\mathrm{DFT}}^{corr}$ correction term will be employed to validate this methodology as explained below, the highly-accurate $V_{\mathrm{CC}}^{corr}$ correction will be used once the validation succeeds.

The correction will be added to the PM7 energy $V_{\mathrm{PM}7}$ so that the PM7 Hamiltonian corrected for non-covalent (nc) interactions would read:

(9) $$V_{\mathrm{PM}7,X}^{nc}=V_{\mathrm{PM}7}+V_{X}^{corr}$$

The strategy of using small representative molecules and sums of two-body functions was successfully employed in the development of intermolecular potentials for interactions of protonated peptides and silyl ions with perfluoroalkane self-assembled monolayers [147,148]. Nevertheless, this strategy will be validated for the new functional groups by running DFT calculations for large systems. This will allow us to compare the DFT-calculated energies with those obtained with $V_{\mathrm{PM}7,\mathrm{DFT}}^{nc}$.

The semiempirical methods, and particularly PM6 and PM7, do not perform well for transition-metal complexes [149]. Our strategy here will be to reoptimize the PM7 Hamiltonian as in previous studies of our group (e.g., see ref. [68]). We will select popular transition metals and ligand molecules used in organometallic catalysis, and will carry out high-level ab initio calculations for our own benchmark database. To gain flexibility in the parametrizations, we will consider the possibility of defining “atom types” for the ligand atoms, depending on the functional groups, in much the same way as that done for the parametrization of the hpCADD NDDO Hamiltonian [150].

### 4.6. Study of Condensed Phase Reactions

Our method is not limited to gas phase reactions. Although currently it only handles reactions in the gas phase, its modular design allows for a smooth adaptation of tsscds to deal with condensed phase reactions. For instance, to study solvent effects, the easiest way would be to use an implicit model, which in practice would only entail adding the appropriate keywords to the templates employed for the different electronic structure programs.

On the contrary, if one wants to use explicit solvent molecules, the MD module must be changed or substituted. At present, the MD module is a modified version of DRC routine in MOPAC2016, which includes different strategies for enhanced sampling, as detailed in the tutorial of tsscds [47]. To include solvent molecules in the MD simulations, one possibility would be to use CHARMM [151] or to adapt DRC. Finally, if the interest is a gas surface reactions, VENUS [152] would be the choice to run the MD simulations because the authors have vast experience using this program.

## Figures and Tables

**Table 1:** Relative product abundances obtained by different computational studies and experiment in the photodissociation of ACRL at 193 nm.

Channel	Chin et al. [96]	Tsscds	Exp [97]
H_2_O	0.01	0.03	0.07
CH_2_O	0.65	0.20	0.07
H_2_	0.09	0.19	0.00
CO	1.00	1.00	1.00
H_2_ + CO + HCCH	6.82	1.49	1.10

**Table 2:** Orders of the hydroformylation reaction with respect to the catalyst and starting materials.

Species	Exp [138]	tsscds [43]	Harvey [133]	Habershon [27]
H_2_	0.6	0.4	0.5	1
CO	<0	<0	<0	<0
catalyst	0.8	0.5	0.5	1
alkene	1	1	1	0.55
